# Pure electronic metal-insulator transition at the interface of complex oxides

**DOI:** 10.1038/srep27934

**Published:** 2016-06-21

**Authors:** D. Meyers, Jian Liu, J. W. Freeland, S. Middey, M. Kareev, Jihwan Kwon, J. M. Zuo, Yi-De Chuang, J. W. Kim, P. J. Ryan, J. Chakhalian

**Affiliations:** 1 Department of Physics, University of Arkansas, Fayetteville, AR 72701, USA; 2 Department of Physics and Astronomy, University of Tennessee, Knoxville, TN 37996, USA; 3 Advanced Photon Source, Argonne National Laboratory, Argonne, IL 60439, USA; 4 Department of Materials Science and Engineering, University of Illinois, Urbana, IL 61801, USA; 5 Advanced Light Source, Lawrence Berkeley National Laboratory, Berkeley, CA 94720, USA

## Abstract

In complex materials observed electronic phases and transitions between them often involve coupling between many degrees of freedom whose entanglement convolutes understanding of the instigating mechanism. Metal-insulator transitions are one such problem where coupling to the structural, orbital, charge, and magnetic order parameters frequently obscures the underlying physics. Here, we demonstrate a way to unravel this conundrum by heterostructuring a prototypical multi-ordered complex oxide NdNiO_3_ in ultra thin geometry, which preserves the metal-to-insulator transition and bulk-like magnetic order parameter, but entirely suppresses the symmetry lowering and long-range charge order parameter. These findings illustrate the utility of heterointerfaces as a powerful method for removing competing order parameters to gain greater insight into the nature of the transition, here revealing that the magnetic order generates the transition independently, leading to an exceptionally rare purely electronic metal-insulator transition with no symmetry change.

One of the greatest challenges of condensed matter physics involves exposing the true underlying mechanisms giving rise to the observed anomalous properties, a situation greatly complicated by the coupling of various interactions, for example competing nematic, structural, and spin transitions in iron pnictide^1^,^2^ or intertwined charge, magnetic, and superconducting order parameters in underdoped high-Tc cuprates^3^,^4^. In strongly correlated electronic materials, the notion of complexity has been synonymous with multiple and often antagonistic ordered phases of intertwined charge, spin, and orbital degrees of freedom^3^,^4^,^5^,^6^,^7^. True insight into the ground state of these materials thus necessitates the ability to selectively eliminate these degrees of freedom to reveal individual contributions.

As a classic case in question, the crossover of an electrically conducting state of a solid into a phase wherein the movement of carriers is prohibited is a prototypical example of such a problem. This metal-to-insulator transition (MIT) is frequently accompanied by emergent order parameters including structural modulation, magnetic, charge, and orbital orderings etc., making it an arduous task to decipher the decisive interaction behind the transition^8^. Despite these complications, metal-insulator transitions have been controllably modified by external stimuli in an effort to disentangle the coupled order parameters to uncover the true progenitor^9^,^10^,^11^,^12^,^13^,^14^,^15^,^16^. Congruent to this effort, a deterministic control over the interfaces between layers with distinct or competing order parameters has further widened the traditional modalities that govern the global phase behavior of correlated electrons^17^,^18^,^19^,^20^,^21^,^22^,^23^,^24^. The heterointerface approach naturally brings forward the important question of whether it is possible to selectively modulate a specific ordering to reveal the primary cause for the phase transition into a multi-ordered ground state. Unlike the previously mentioned efforts, where the system comes back to the original ground state when the external stimulus is removed, the present study undertook to suppress order parameters by the virtue of epitaxial stabilization, effectively freezing the system in an atypical state.

Specifically, a 15 unit cell thin film of rare-earth nickelate NdNiO_3_ (NNO) is utilized as a model system exhibiting a first-order MIT that in the bulk involves structural, charge, and antiferromagnetic order parameters whose entanglement has obscured true understanding of the mechanism underpinning the transition, Fig. 1A,C 6,7,25,26,27,28,29,30,31,32,33,34,35,36,37,38. Interestingly, recent work by Hepting and Wu *et al.* has shown that superlattices under compressive strain utilizing PrNiO_3_ and PrAlO_3_ can suppress the MI- and charge order (CO) transitions, while preserving the AFM transition, leading a rare metallic AFM state^28^,^29^. These considerations suggest some connection with the magneticly driven Slater transition, found experimentally for the first time recently^39^,^40^,^41^,^42^,^43^. However, several key differences between the nickelates and a pure Slater transition are evident, namely the first-order nature of the MIT and the large spectral weight transfer^6^,^44^. These factors indicate a Mott-like nature of the MIT, but the commensurate magnetic transition also rules out a true Mott MIT^44^,^45^,^46^. Our experiment, spanning x-ray absorption spectroscopy (XAS) and resonant x-ray scattering (RXS), demonstrates that in the ultra thin limit for films the MIT persists with the same bulk-like E′- antiferromagnetic ordering and changes in electronic structure while the charge order parameter, and accompanying structural transition, are completely removed at all temperatures. These findings imply the exceptional case of an isosymmetric and purely electronic metal-insulator transition, as seen in some Mott transitions^15^,^47^, driven by both strongly correlated electrons and magnetic ordering and is in sharp contrast to the present understanding of the physics of rare-earth nickelates^34^,^35^,^36^,^37^.

## Electronic and Magnetic Configuration

Reduction of the degrees of freedom through heterostructuring presumably alters the electronic structure from it’s bulk-like state. Indeed, in nickelates, thin-film geometry and proximity to the interface has been shown to strongly alter the electronic structure of the constituent layers and, thus, requires investigation; numerous XAS reports detail the change of the electronic structure across the MIT showing a characteristic splitting of the Ni L_3_ edge below the MIT into two distinct peaks and a narrowing of the *d*-electron bandwidth in the insulating state^6^,^7^,^25^,^32^,^48^,^49^. These two distinct effects are the spectroscopic signatures of the stabilization of an insulating state in the nickelates. As seen in Fig. 2A, in the case of ultra thin films of NNO, the Ni L_3_ edge does indeed show a clear splitting below the MIT, with a line shape that clearly indicates the stabilization of the Ni^3+^ state^6^,^25^,^32^. Tracking the intensity in between the two peaks (inset of Fig. 2A) confirms a distinct spectroscopic change quantitatively very close to bulk-like behavior across the MIT^49^. Similarly, the O K-edge pre-peak reflects the band narrowing across the MIT, Fig. 2B; the sudden shift in bandwidth is commensurate with the onset of the insulating state at ~150 K, (Fig. 3A, solid lines)^25^. This prepeak arises due to hole doping of the O sites and is an important element in charge transfer insulators, with early work showing the importance for RNO^50^.The first-order nature of the transition is evident by the thermal hysteresis of the MIT^6^,^7^,^25^,^28^,^34^. Thus, both XAS and transport measurements affirm that the ultra thin structural motif does not generate any anomalous electronic structure effects across the MIT, making it an ideal candidate for investigation of the commensurate order parameters.

**Figure 1:**
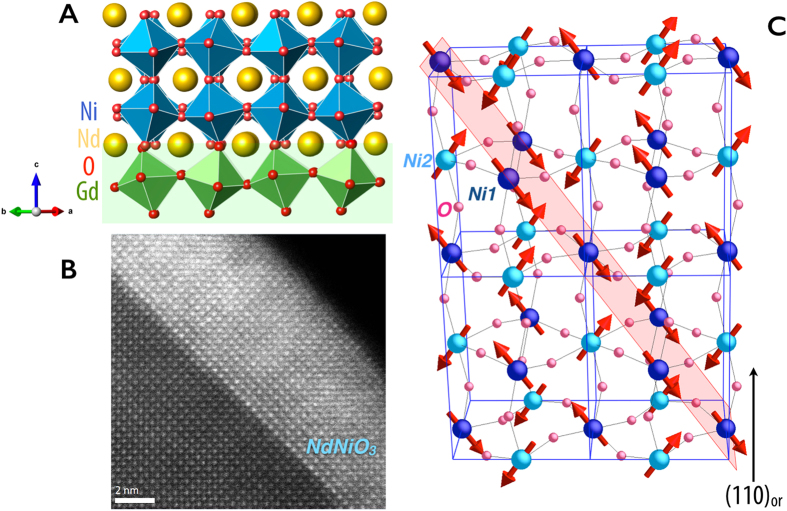
(**A**) Heterostructure interface of NNO grown on NGO. (**B**) TEM showing atomically sharp interface. (**C**) E′-type antiferromagnetic ordering in the nickelates with the (111)_*pc*_ plane highlighted. The dark and light blue spheres represent the nickel sites with charge of 3 ± *δ*^52^,^53^.

**Figure 2:**
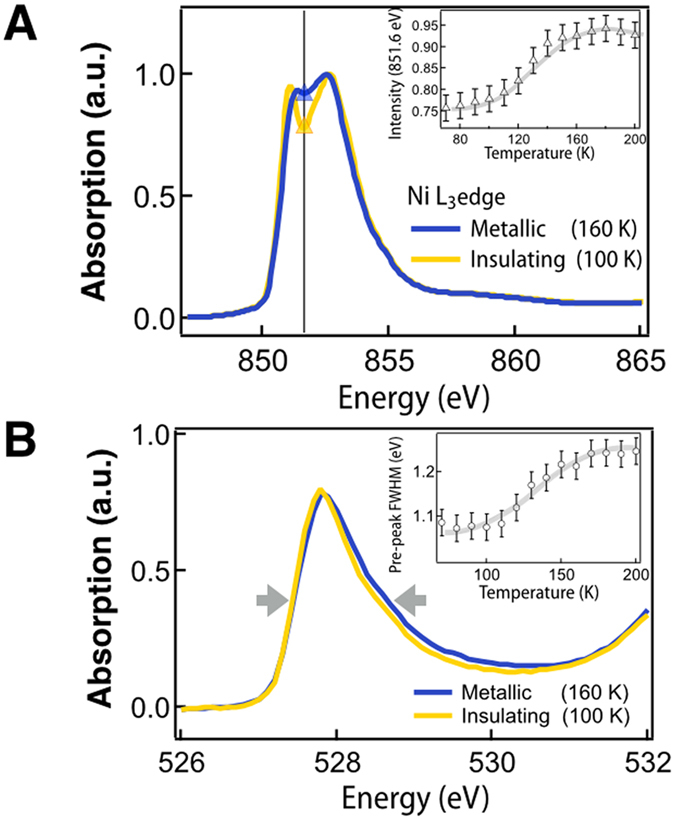
(**A**) XAS at the Ni L_3_-edge for the metallic and insulating states. Inset shows the intensity between the Ni^3+^ and multiplet peaks, highlighting the sudden narrowing of the peaks across the MIT. (**B**) XAS at the O K-edge for the same. Inset shows the change in the FWHM, arrows, of the O prepeak showing the bandwidth narrowing. All hatched lines are guides to the eye.

**Figure 3:**
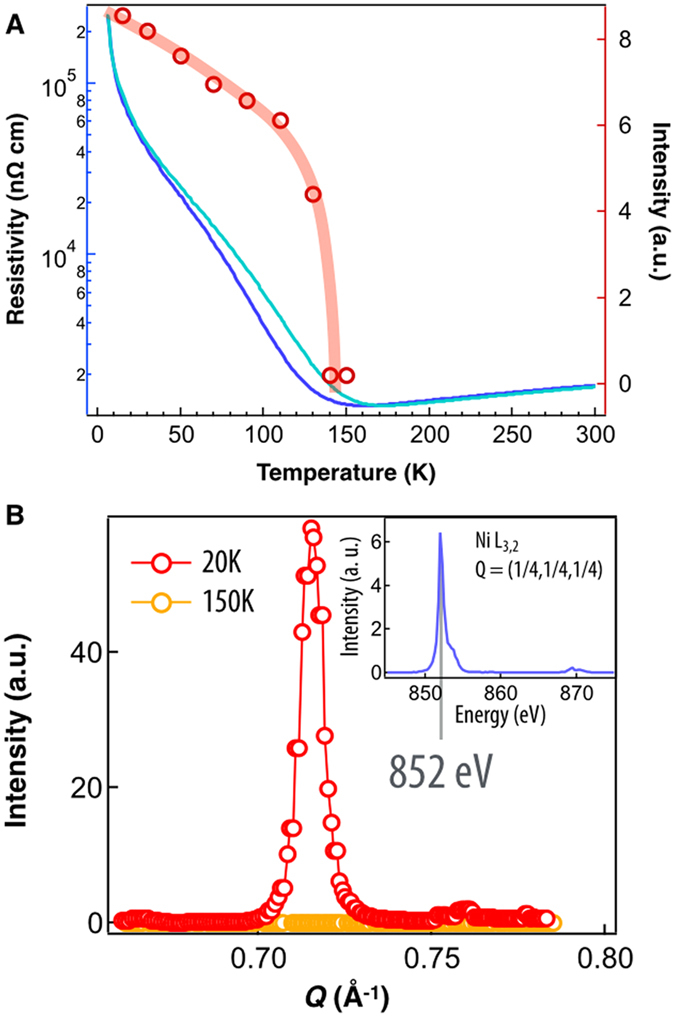
(**A**) Left axis: Temperature dependence DC transport for cooling (blue) and warming (cyan) cycles showing a strong hysteresis typical of the first-order MIT. Right Axis: Temperature dependence of the forbidden Bragg peak intensity corresponding to the magnetic order parameter. (**B**) Low and high temperature magnetic Bragg peak corresponding to E′-type anti-ferromagnetism. The inset shows the resonant energy scan at the Ni L_3_ and L_2_ at the peak at 20 K.

Spin ordering is a prevalent ingredient in Mott transitions^8^,^51^. In fact, Mott transitions feature local moments both above and below the MIT, as evidenced for these films in the supplementary information. In the nickelates, the magnetic ordering has received widespread attention due to the unusual stacking of ferromagnetic planes along the (1 1 1)_*pc*_ (pc = pseudo-cubic) direction that are coupled antiferromagnetically (AFM) to one another in an up-up-down-down pattern, a non-collinear periodic behavior, and a magnetic unit cell consisting of four structural unit cells, shown in Fig. 1C 52,53. Probing this anomalous, E′- AFM ordering in ultra-thin film geometry is quite challenging; Fig. 3A displays the results of the soft x-ray resonant scattering (RXS) at the (1/2 0 1/2)_*or*_ (or = orthorhombic) reflection with the energy tuned to the Ni L_3_ edge (852 eV) below the MIT. This structurally forbidden Bragg reflection corresponds to a 4-fold unit cell repetition in the (1 1 1)_*pc*_ direction. As seen in Fig. 3A, circles, the intensity of this reflection tracks very close with the MIT, suddenly rising above the background noise at around 140 K and steadily increasing until beginning to stabilize at low temperature. Both the periodicity and spectroscopic signature are in excellent agreement with previous studies on thick NdNiO_3_ films and bulk powders^52^,^54^. In short, these results show that the bulk E′-type AFM order parameter is preserved and conforms with the MIT despite bi-axial strain (~1.4%). With the expected electronic structure response (i.e. AFM order parameter, and first-order MIT) the pinning of the lattice to the substrate does not cause any anomalous perturbation to the bulk-like magnetic and transport behavior of the nickelate film. In addition, for any bulk rare-earth nickelate, the insulating ground state is characterized by the presence of CO and the structural transition from the orthorhombic *Pbnm* symmetry of the metallic phase to monoclinic *P*2_1_/*n* symmetry of the insulating phase^6^,^7^,^35^.

## Probing Lattice Symmetry and Charge Ordering

First, we discuss the issue of lattice symmetry transformation, which is considered to be critical for the MIT. Heterostructuring naturally leads to a modulation of the film lattice due to the strong bonding with the substrate’s ions. When the film becomes thick enough the relaxation of elastic strain is inevitable and effectively decouples the film from the substrate^55^. In the ultra thin regime, however, the film is pinned to the substrate with no detectable relaxation and the heteroepitaxy infact controls the lattice degrees of freedom therein^32^,^48^.

**Figure 4:**
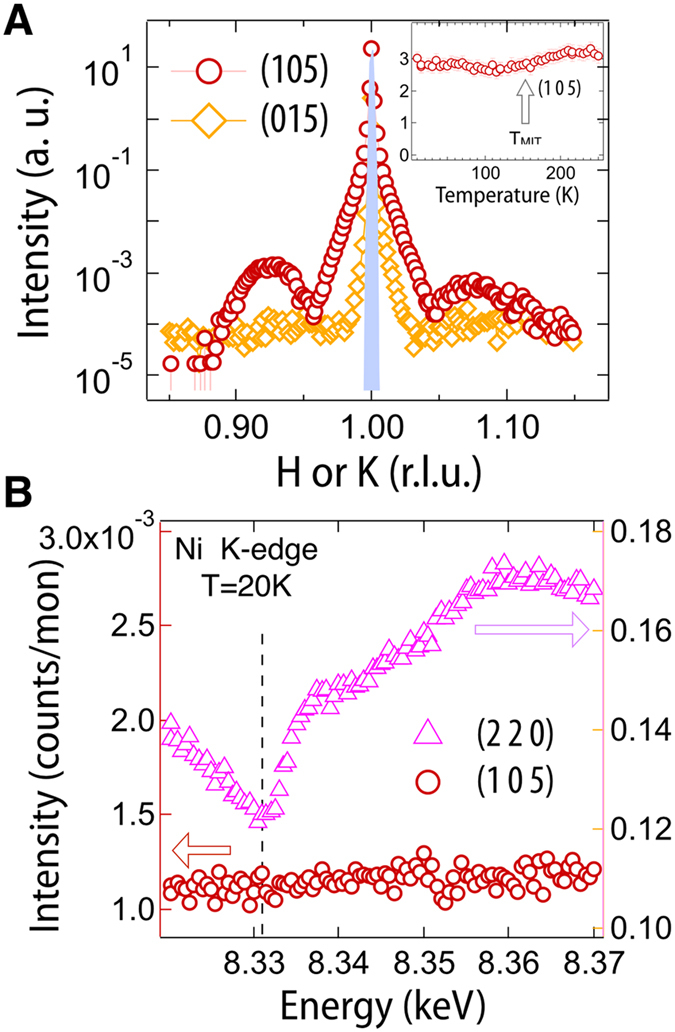
(**A**) Scattering around the (1 0 5)_*or*_ and (0 1 5)_*or*_ peaks at low temperature ~10 °K (the sharp peak at 1.00 is the substrate). The inset show the measured intensity of the (1 0 5)_*or*_ peak for several temperatures crossing the MIT. (**B**) Ni K-edge resonance scans at the (1 0 5)_*or*_ and (2 2 0)_*or*_ peaks.

*Pbnm* (metal) and *P*2_1_/*n* (insulator) space groups have the same Ni arrangement, however they are split into different Wycoff positions with the symmetry lowering. These inequivalent Ni sites carry a rock-salt pattern of charge disproportionation Ni^3±*δ*^ (purely ionic picture) giving rise to the CO parameter. In recent years it has been found that, while hard RXS is a powerful tool for investigating charge ordering, careful analysis is required to avoid the misinterpretation of CO for small distortions of the oxygen octahedral network^34^,^35^,^56^. With this caution in mind, we investigated the (0 1 5)_*or*_ and (1 0 5)_*or*_ reflections, which are conventionally used to probe the lowering of the symmetry to monoclinic *P*2_1_/*n*, Fig. 4A,B 34,35,56,57,58. The (105)_*or*_ and (015)_*or*_ peaks were chosen as they were shown to be quite sensitive to Ni CO in numerous previous studies^34^,^35^,^36^,^37^.

Figure 4A displays scans along the L reciprocal space vector (L-scan) at the (0 1 5)_*or*_ and (1 0 5)_*or*_ peaks. The (1 0 5)_*or*_ peak is symmetry allowed for orthorhombic NNO, as a Bragg peak corresponds to the Nd sublattice, thus the film peak with Kiessig fringes is anticipated. As expected above the CO transition, this (105) reflection should have no Ni contribution until the charge ordering breaks the *Pbnm* symmetry in the low temperature insulating phase; the CO then leads to an additional contribution to the peak from Ni causing a sharp change in signal strength, especially when the x-rays are tuned to the resonant Ni K-edge (8.34 keV)^35^. Surprisingly, as the temperature is scanned across the MIT, no detectable change in the peak intensity is observed, Fig. 4A inset. With an intensity error of approximately 5%, this implies an upper limit on CO below our detection limit of 2*δ* = 0.073 ± 0.007e, insignificant compared to the 2*δ* = 0.45 ± 0.04e found by Staub *et al.*^34^. This result immediately implies that neither charge ordering nor the associated symmetry breaking occurs across the transition. Lending additional evidence, the (0 1 5)_*or*_ peak, which is symmetry-forbidden for *Pbnm*, does not appear at any temperature, thus confirming the isosymmetric nature of the MIT^34^.

Furthermore, a key feature of resonant scattering is that the additional terms within the scattering factor are *highly* sensitive to the x-ray energy around an absorption edge^59^,^60^. Figure 4B shows the energy scan at the (1 0 5)_*or*_ peak which further corroborates the above picture with higher sensitivity, confirming that *no Ni resonance signal (i.e. symmetry lowering) is detected below the MIT*. This is in stark contrast to all previous reports on both thick films and bulk where strong, temperature dependent resonance was shown to track with the MIT^34^,^35^,^36^,^37^. To further verify this finding, the allowed (2 2 0)_*or*_ reflection was measured and shows the expected Ni resonance signal, confirming that the Ni contribution is certainly detectable in our experimental setup. These results confirm that across the MIT (*i*) no bond disproportionation of NiO_6_ occurs and the metallic phase *Pbnm* lattice symmetry is preserved^35^,^36^, and (*ii*) since no detectable Ni resonance is observed no charge ordering emerges in the insulating phase. These findings imply that the ultra thin films have stabilized a previously unknown nickelate ground state consisting of an insulating orthorhombic phase with AFM order. However, ultra thin films of the more strongly distorted EuNiO_3_ on NGO substrates displayed bulk-like CO, suggesting this anomalous state occupies only a narrow band of phase space^61^. Intriguingly, as observed here, the case of a phase transition without a structural symmetry change can only be first order and is exceptionally rare in complex materials, with the most prominent examples being analogous to the liquid-gas transformation^51^,^62^. For complex oxides, to our best knowledge, there are only two known cases of this type of MIT, i.e. Cr-doped V_2_O_3_^15^ and the surface driven Ca_1.9_Sr_0.1_RuO_4_^47^. It should be noted, experiments have also reported evidence of photoinduced MITs without resolved structural transitions^63^,^64^. Finally, these measurements are sensitive to long range ordering and do not rule out the possibility of a short-range charge ordered state on Ni or bond-centered ordering^65^,^66^.

## Theoretical Presage

Driven by the heterointerface, CO removal and the stabilization of the unknown Mott phase within this class of materials is of great interest and yet has some precedent in past theoretical work^38^,^67^,^68^,^69^,^70^,^71^,^72^,^73^,^74^. For example, two recent studies utilizing different theoretical methods by Lee *et al.*^67^,^68^ have proposed that the CO is slaved to the *E*′-magnetic ordering in the weak coupling limit, and can indeed disappear under certain conditions; in particular, using Landau theory, the theory suggests that restricting the nickelates to the ultra-thin film regime may remove the CO. On the other hand, the predicted phase changes the *Q*-vector for the antiferromagnetic ordering, which is in variance with the experiment. Beyond this, Park *et al.* have demonstrated that within dynamical mean field theory (DMFT), despite the near Fermi-energy imbalance in the spectral weight between the two Ni sites, the total valence of Ni on both sites is practically identical, with the two different Ni sites instead hybridizing with O, leading to an S = 1 state on the larger octahedra (3d^8^) and an S = 0 state formed due to AFM coupling with the O holes (3d^8^)^73^,^74^. However, when the lattice symmetry is raised to *Pbnm* a metallic state with no MIT has emerged. More recently, Johnston *et al.*^72^ utilized Hartee-Fock methods to show the NiO_6_ octahedra form an alternating pattern of collapsed and expanded octahedra, giving a *d*^8^ + *d*^8^L^2^ state, where no CO on Ni occurs.

Finally, using LSDA + U calculations, Yamamoto *et al.*^38^ obtained results that are in the good agreement with our observations. Specifically, the calculated electronic and magnetic structure in orthorhombic NNO is found to be an insulating state with no Ni CO (as expected for equivalent Ni sites in *Pbnm* symmetry). In addition, the calculation shows that the magnetic space group is lowered to monoclinic due to different spin density polarizations around two O sites that preserve the equivalence of Ni sites in the *Pbnm* space group. Most importantly, this symmetry breaking state involving holes on oxygen and driven by the Hubbard U, opens an insulating gap, which agree well with the previous work^44^. At this point we can conjecture that while in the bulk structural symmetry is indeed lowered to *P2*_1_*/n,* the epitaxial interface is able to preserve the orthorhombic structural symmetry of the metallic phase. The resulting ground state, observed experimentally, can be obtained within the LSDA + U framework, supporting the notion that the bulk-like MIT and magnetic order parameter can be attained with the charge and structural order parameters removed. In this work, we find the heterointerface acts as a powerful tool to effectively isolate the magnetic order parameter, which drives the bulk-like MIT independently.

## Concluding Remarks

In conclusion, the reduction of the number of simultaneously competing order parameters commensurate with a phase transition from a metallic to an insulating state with both Mott and Slater characteristics has been achieved on a prototypical ultra thin film of NNO. The thin film heteroepitaxy prevents symmetry lowering from *Pbnm* to *P2*_1_*/n* across the MIT, thus removing the bulk-like CO parameter. Despite this anomalous state, the MIT persists with no significant effect on the magnetic order parameter. The magnetic order parameter is identified as the culprit which drives the pure electronic MIT in the nickelates, highlighting the utility of this emerging method to sunder the competing order parameters. Our findings suggest that application of this method to eliminate specific order parameters to highly entangled or “hidden” orders found in cuprates, pnictides, heavy fermions and chalcogenides families may shed new light on their anomalous ground states^2^,^75^.

## Methods

Epitaxially stabilized ultra thin (15 uc) films of NdNiO_3_ on (110)_*or*_ oriented NdGaO_3_ substrates grown by pulsed laser deposition, various other techniques have confirmed the high quality of these films^32^. This orientation stabilizes the NNO films with the psuedo-cubic (pc) c-axis oriented in the growth direction. To elucidate the electronic structure, x-ray absorption spectroscopy was measured at the 4-ID-C beam line of the Advanced Photon Source at Argonne National Laboratory. In order to investigate whether the bulk-like E′-type anti-ferromagnetic ordering occurs in these ultra thin films, systematic resonant soft x-ray scattering experiments were performed on the Ni L_3_ edge at the (1/2 0 1/2)_*or*_ forbidden Bragg reflection at several temperatures traversing the MIT at the BL8 beam line of the Advanced Light due to the drastic enhancement of the magnetic scattering cross sections for transition metals’ L-edges^59^,^60^. Data was taken with a two-dimensional detector with the peak area integrated. In contrast to this, to probe the charge ordering in these systems higher order reflections, such as the (1 0 5)_*or*_, were measured with resonant hard x-ray scattering at the Ni K-edge at the 6-ID-B and 33-BM beam lines of the Advanced Photon Source due to the suitable wavelengths for diffraction from crystals^34^.

## Additional Information

**How to cite this article**: Meyers, D. *et al.* Pure electronic metal-insulator transition at the interface of complex oxides. *Sci. Rep.* **6**, 27934; doi: 10.1038/srep27934 (2016).

## Supplementary Material

Supplementary Information
