# Supplementary material for: Pure electronic metal-insulator transition at the interface of complex oxides
Source: Sci Rep. 2016 Jun 21;6:27934. doi: 10.1038/srep27934 (PMC4914986; doi:10.1038/srep27934)
Supplement: Supplementary Information [file srep27934-s1.pdf]

Supplementary information: Pure electronic metal-insulator transition at the interface of complex oxides

D. Meyers[1,*], Jian Liu[2,*], J. W. Freeland[3], S. Middey[1], M. Kareev[1], Jihwan Kwon[4], J. M. Zuo[4], Yi-De Chuang[5], J.-W. Kim[3], P. J. Ryan[3], and J. Chakhalian[1]
[1]*Department of Physics, University of Arkansas, Fayetteville, AR 72701, USA*
[2]*Department of Physics, University of California, Berkeley, CA 94720, USA*
[3]*Advanced Photon Source, Argonne National Laboratory, Argonne, IL 60439, USA*
[4]*Department of Materials Science and Engineering, University of Illinois, Urbana, IL 61801, USA*
[5]*Advanced Light Source, Lawrence Berkeley National Laboratory, Berkeley, CA 94720, USA and*
[*]*Both authors contributed equally; E-mail: dmeyers@uark.edu, jian.liu@berkeley.edu*

SFig. 1 displays X-ray magnetic circular dichroism (XMCD) measurements above and below the metal insulator transition for a 15 unit cell $NdNiO_3$ film on $NdGaO_3$ [1]. The lack of a significant change indicates a similar local moment on Ni in both the metallic and insulating states, as required for a Mott transition.

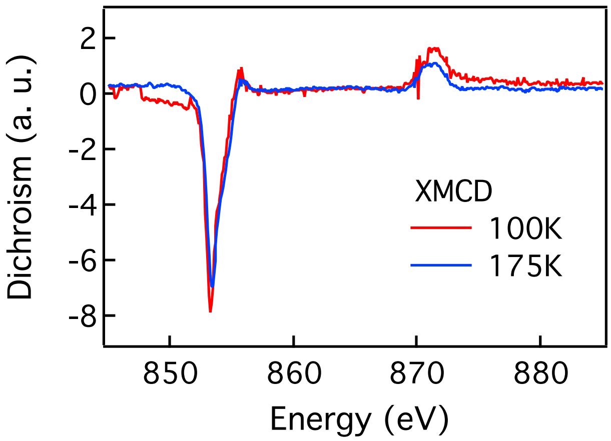

FIG. 1: (Color online) XMCD at the Ni $L_{3,2}$-edges.

[1] C. T. Chen *et al*. Experimental Confirmation of the X-Ray Magnetic Circular Dichroism Sum Rules for Iron and Cobalt. *Phys. Rev. Lett* **75**, 152-155 (1995).
